# The Intestinal Bacterial Microbiome and *E. histolytica* Infection

**DOI:** 10.1007/s40475-016-0083-1

**Published:** 2016-06-30

**Authors:** Stacey L. Burgess, William A. Petri

**Affiliations:** Department of Medicine, Division of Infectious Diseases and International Health, University of Virginia Health System, Charlottesville, VA USA

**Keywords:** *Entamoeba histolytica*, Microbiota, Trained immunity, Amebiasis host pathogen interactions

## Abstract

*Entamoeba histolytica,* the etiological agent of amebiasis, is a significant cause of pediatric diarrhea in South Asia and sub-Saharan Africa. The clinical outcome of an *E. histolytica* exposure varies enormously and can present as diarrhea, dysentery, or amebic liver abscess. Host and parasite factors likely contribute to the outcome of infection with the parasite, but do not explain the wide variation in presentation of disease. This suggests that other environmental factors affect disease. An emerging body of work suggests that the host intestinal bacterial microbiome may have a significant influence on the development and outcome of amebiasis.

## Introduction

It is estimated that 1 million children under the age of five die from diarrhea each year. Population-based surveys of pediatric diarrheal diseases have linked *Entamoeba histolytica,* the etiological agent of amebiasis, to a significant number of diarrheal cases in South Asia and sub-Saharan Africa [1•]. The outcome of an *E. histolytica* exposure varies tremendously and can present as diarrhea, dysentery, or amebic liver abscess, with liver disease occurring primarily in men [2]. Disease occurs relatively infrequently however, and many cases of exposure are asymptomatic. Host and parasite factors likely contribute to the outcome of infection with the parasite [3, 4]. However, these factors do not fully explain the wide variation in presentation in patients. Therefore, an environmental factor likely contributes to the progression of *E. histolytica* infection.

**Fig. 1:**
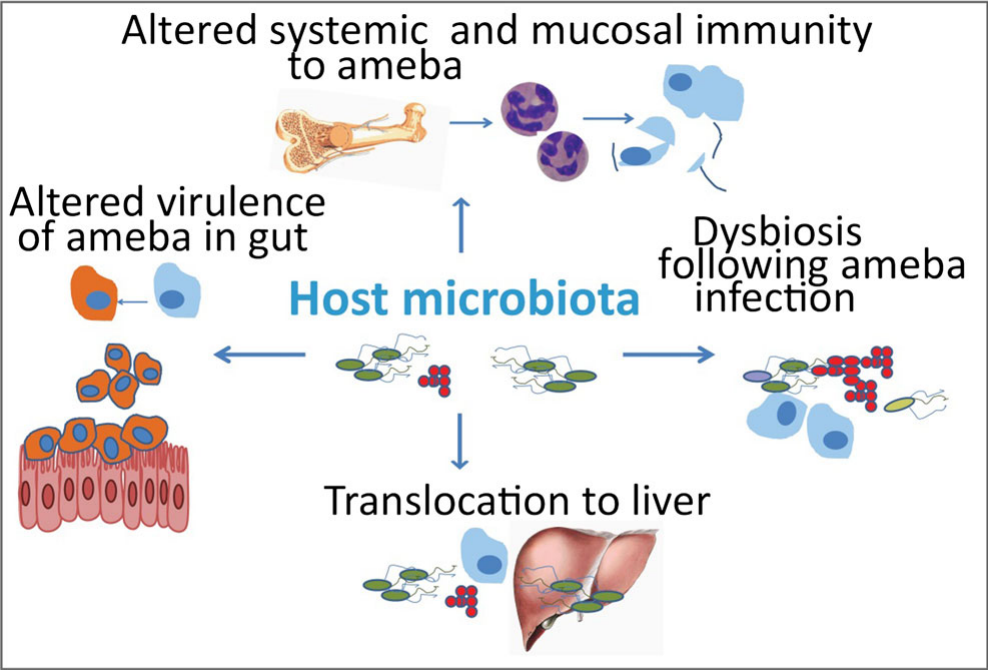
Potential interactions between the microbiota and *E. histolytica*. The intestinal bacterial microbiota may influence the virulence of the ameba and its ability to colonize the gut (*left*), as well as the host’s immune response at baseline, and during amebiasis (*top*). In turn, the ameba infection may influence the composition of the microbiota, resulting in dysbiosis (*right*), and may alter localization of the intestinal microbiota (*bottom*). These factors may in turn influence the clinical outcome of *E.histolytica* infection

Initial infection with *E. histolytica* occurs after ingestion of faecally contaminated water or food containing *E. histolytica* cysts. These cysts then undergo excystation in the lumen of the small intestine. The trophozoite stage of the ameba then feeds on resident bacteria as well as the intestinal mucosa in severe cases. Extraintestinal amebiasis occurs when the ameba invades the intestinal mucosa and travels to the blood stream [3, 5]. This manifests as liver or brain disease. In each case however, the initial interaction of the ameba with the host occurs in the context of the bacterial microbiota [6, 7•]. The microbiota, including the normal flora of the human gastrointestinal tract, is a complex community of bacteria that is composed of at least several hundred species. Ultimately, there are considerably more bacterial cells than there are eukaryotic cells in the human body and these organisms form a symbiosis that influences human physiology and disease progression [5, 8, 9]. This community of bacteria may have a significant influence on the virulence of the amoeba itself, its ability to colonize the gut, and the host’s immune response at baseline, and during amebiasis. The bacterial microbiota is therefore a significant environmental factor that may influence the clinical presentation and outcome of *E. histolytica* infections (Fig. 1).

## The Microbiota and *E. histolytica* Virulence

Many factors contribute to virulence in *E. histolytica* infection. This includes parasite factors, such as surface lectins that bind oligosaccharides on intestinal mucins [10], and host factors such as genetic background, including polymorphisms in the leptin receptor [11]. Coinfections with enteropathogens, and nutritional state, particularly malnutrition, also influence disease [1•, 10]. Marie et al. has recently provided an in depth review of these, and other factors, that influence *E. histolytica* virulence in human hosts and models of disease [7•]. Studies in gnotobiotic animals have also indicated that commensal bacteria are necessary for virulent *E. histolytica* infection [12, 13]. A recent study by Reyna-Fabián et al has also suggested that the host intestinal microbiota may influence the parasite during one of the more severe forms of parasite infection, amebic liver abscesses (ALA) [14].

Reyna-Fabián et al. utilized 16S ribosomal RNA (rRNA) gene sequencing to identify bacterial species and query bacterial population diversity and phylogenetic relationships between organisms in liver aspirates from patients with ALA as well as pyogenic liver abscess (PLA). They compared this with clinical data that included the etiology (pyogenic, amebic, or mixed) of the hepatic abscess as well as *Entamoeba* genotypes and then performed statistical tests for significant associations. Their results suggested that a high percentage of amebic liver abscesses are co-infected with non-cultivable bacteria of the intestinal microbiota, as well as potentially pathogenic bacteria. However, they were not able to identify specific groups of bacteria associated with any species or genotype of *E. histolytica* or *E. dispar* in ALA [14]. Future studies with larger population sizes may allow determination of which components of the intestinal bacterial microbiota might correlate with ALA. This work suggests however that the intestinal bacterial microbiota may interact with *E. histolytica* at extraintestinal sites, perhaps via translocation with the ameba.

## *E. histolytica* and the Composition of the Intestinal Microbiota in Human Populations

Probiotics and commensal bacteria have been suggested to have some influence on the outcome of protozoan infections [15•]. However, very few studies have specifically examined associations between *Entamoeba* infection and variation in the human intestinal microbiota. *E. histolytica* colonization may alter, or be influenced by, the ratio of bacterial phyla present in the host microbiota. Verma et. al. demonstrated that *E. histolytica* associated dysbiosis in northern India was characterized by significantly less *Clostridia, Bacteroides*, *Lactobacillus*, *Campylobacter,* and *Eubacterium* and increased *Bifidobacterium* species in stool samples as compared to healthy controls [16]. Commensal bacteria and components of the microbiota generally exist in symbiosis with the host; however, some normal flora have the potential to cause, or exacerbate, existing disease. These commensals with pathogenic potential are known as pathobionts [17].

Recently, Morton et al. characterized the fecal microbiota of rural populations in southwest Cameroon via 16S rRNA gene sequencing and determined *Entamoeba* colonization via microscopy [18••]. The use of microscopy however does not allow discrimination between intestinal amoeba *E. dispar,* which is generally nonpathogenic, and *E. histolytica,* the causative agent of amebiasis [19, 20]. They discovered that *Entamoeba* colonization is significantly correlated with microbiome composition and diversity. Most interestingly, they show that colonization by the amoeba can be predicted based on the composition of an individual’s gut microbiota with 79 % accuracy. Several of the taxa most important for distinguishing *Entamoeba* presence, which included *Prevotella copri*, are also associated with autoimmune diseases [21]. *P. copri* can exist as a normal component of the gut microbiota, but is also a pathobiont, and is associated with more severe inflammation in several diseases [22]. This highlights the potential influence of inflammation driven by the gut microbiome in altering amoeba infection.

Gilchrist et al. have just prospectively studied the natural history of *E. histolytica* colonization and diarrhea in infants in an urban slum located in Dhaka, Bangladesh. In this population, approximately 80 % of children were infected with *E. histolytica* by 2 years of age [23••]. High parasite burden and expansion of *P. copri* was associated with diarrhea. This work suggested that specific components of the microbiota and pathobionts might be associated with symptomatic or asymptomatic *E. histolytica* colonization. These studies, and future microbiome studies, provide for a better understanding of environmental factors that underlie the wide variation in clinical presentation of *E. histolytica* infection. However, it is difficult to test causality in population-based studies; therefore, disease models provide a useful tool to understand how the microbiome may influence the progression and severity of ameba infection.

## Microbiota Modulation of the Immune System and Potential Influence on *E. histolytica* Infection

The intestinal bacterial microbiota has recently been shown to be important in modulating bone marrow processes that give rise to immune effector cells needed for pathogen clearance, such as neutrophils and inflammatory macrophages, and in providing protection from enteric infection [24, 25]. Several studies have also suggested that intestinal infection with one organism, or vaccination, may persistently alter innate immune populations to provide protection from infection with unrelated pathogens. This idea has been referred to as trained immunity [26, 27•]. However, the mechanism of how unrelated organisms might generate this innate memory to provide protection is not currently well understood. Murine models of amebiasis provide a method to test how the intestinal microbiota may influence innate immune populations and subsequent infection with the parasite [28].

Recently, we have shown that mice colonized with the commensal *Clostridia*, segmented filamentous bacteria (SFB), are protected from experimental amebiasis. Bone marrow derived dendritic cells (BMDCs) from SFB colonized mice produced significantly higher levels of interleukin 23 [29••]. IL-23 is a cytokine [30] linked to induction of IL-17 and neutrophils, which in turn have been shown to be important in immunity to the ameba [31, 32]. Transfer of BMDCs derived from mice colonized with SFB provided protection from *E. histolytica* infection. A host damage associated molecular pattern molecule serum amyloid A (SAA) was also increased in the serum of SFB colonized mice compared to mice lacking the commensal. Treatment of BMDCs with SAA partially recapitulated the effect of increased Interleukin 23 that was observed in BMDCs from SFB colonized mice [29••]. This work suggested that gut colonization with a commensal *Clostridia* might alter bone marrow cells and that host factors induced by the microbiota, such as SAA, might alter the course of *E. histolytica* infection.

Therefore, trained immunity may provide another potential pathway by which components of the bacterial intestinal microbiota could influence the outcome of amebiasis. Gut commensals or pathobioants may serve to alter the immune system in such a way that infection with *E. histolytica* results in a more robust mucosal immune response. This robust response could represent a doubled-edged sword; on one hand, it may help to clear the ameba, as in our murine model, or it might contribute to intestinal damage and more severe pathology and colitis in patients.

## Conclusions

Recent population-based and murine studies have highlighted the importance of the intestinal bacterial microbiome and *E. histolytica* infection. The microbiome and parasite may interact in various ways, which may include alteration of virulence of the ameba, perhaps both in gut infections and in liver abscesses, induction of colonization resistance, or dysbiosis induced by the ameba, and modulation of host immunity, which alters the outcome of parasite infection. Many questions remain however. It is not truly understood how *E. histolytica* affects the composition of microbiota, or how the microbiota in turn may influence the progression and severity of amebiasis. Further exploration of interactions between the gut microbiome and *E. histolytica* will thus help provide tools and approaches that will help in the diagnosis and treatment of amebiasis.
